# A Consistent Orally-Infected Hamster Model for Enterovirus A71 Encephalomyelitis Demonstrates Squamous Lesions in the Paws, Skin and Oral Cavity Reminiscent of Hand-Foot-and-Mouth Disease

**DOI:** 10.1371/journal.pone.0147463

**Published:** 2016-01-27

**Authors:** Win Kyaw Phyu, Kien Chai Ong, Kum Thong Wong

**Affiliations:** 1 Department of Pathology, Faculty of Medicine, University of Malaya, Kuala Lumpur, Malaysia; 2 Department of Biomedical Science, Faculty of Medicine, University of Malaya, Kuala Lumpur, Malaysia; University of Minnesota College of Veterinary Medicine, UNITED STATES

## Abstract

Enterovirus A71 (EV-A71) causes self-limiting, hand-foot-and-mouth disease (HFMD) that may rarely be complicated by encephalomyelitis. Person-to-person transmission is usually by fecal-oral or oral-oral routes. To study viral replication sites in the oral cavity and other tissues, and to gain further insights into virus shedding and neuropathogenesis, we developed a consistent, orally-infected, 2-week-old hamster model of HFMD and EV-A71 encephalomyelitis. Tissues from orally-infected, 2-week-old hamsters were studied by light microscopy, immunohistochemistry and *in situ* hybridization to detect viral antigens and RNA, respectively, and by virus titration. Hamsters developed the disease and died after 4–8 days post infection; LD_50_ was 25 CCID_50_. Macroscopic cutaneous lesions around the oral cavity and paws were observed. Squamous epithelium in the lip, oral cavity, paw, skin, and esophagus, showed multiple small inflammatory foci around squamous cells that demonstrated viral antigens/RNA. Neurons (brainstem, spinal cord, sensory ganglia), acinar cells (salivary gland, lacrimal gland), lymphoid cells (lymph node, spleen), and muscle fibres (skeletal, cardiac and smooth muscles), liver and gastric epithelium also showed varying amounts of viral antigens/RNA. Intestinal epithelium, Peyer’s patches, thymus, pancreas, lung and kidney were negative. Virus was isolated from oral washes, feces, brain, spinal cord, skeletal muscle, serum, and other tissues. Our animal model should be useful to study squamous epitheliotropism, neuropathogenesis, oral/fecal shedding in EV-A71 infection, person-to-person transmission, and to test anti-viral drugs and vaccines.

## Introduction

Enterovirus A71 (EV-A71) is a non-enveloped, single-stranded, positive-sense RNA virus which belongs to the human *Enterovirus* A species group within the *Picornaviridae* family. The EV-A71 genome is approximately 7.4 kb and encodes for 4 capsid proteins and other non-structural proteins [1,2]. It is one of the enteroviruses most often associated with large outbreaks of pediatric hand-foot-and-mouth disease (HFMD) [3,4]. Classical HFMD presents with skin rashes or vesicles on the palms, soles, knees and buttocks, and vesicles or ulcers in the oral cavity and tongue [5]. Although most patients recover uneventfully, EV-A71 infection may sometimes be complicated by aseptic meningitis, acute flaccid paralysis and encephalomyelitis [6–8].

Person-to-person transmission of enteroviruses, most commonly occurs through fecal-oral and oral-oral routes [1]. Consistent with this, the primary replication sites for the virus could be in the oral cavity and/or gastrointestinal tract as virus was frequently isolated from throat swabs, oral secretions and stools [1,9–11]. More recently, the tonsillar crypt epithelium has been identified as an important extra-central nervous system (CNS) viral replication site and a source of virus shedding into the oral cavity, and may also be a portal for viral entry into the body [12]. In complicated HFMD, neuroinvasion most probably follows viremia [13–15]. Fatal cases of EV-A71 encephalomyelitis showed stereotyped distribution of inflammation in the spinal cord, brainstem, hypothalamus, cerebellar dentate nucleus and the cerebrum [16,17]. Virus could be isolated from CNS tissues, and viral antigens/RNA and virions were localized to infected neurons, confirming viral cytolysis as an important cause of neuronal injury [16–18].

Viral predilection for neurons or neuronotropism has also been demonstrated in monkey and mouse models of EV-A71 infection [13,19–23]. Furthermore, retrograde axonal viral transport up peripheral and cranial motor nerves to infect the CNS has also been shown in the mouse model [21,24]. However, in most of these models, the routes of infection were mostly parenteral, via intraspinal, intracerebral, intratracheal, intraperitoneal, intramuscular, and subcutaneous routes [13,20,21,24]. Although infection by the natural oral route in animal models is desirable, it is rare and consistently successful infections have never been described [21,22,25,26]. Preliminary observations in a new hamster model used to test the protective efficacy of an EV-A71 candidate vaccine against infection by a mouse-adapted virus (MAV), suggested that it may be useful as an alternative small animal model for EV-A71 infection [27]. In this study, we report its further characterization as a suitable model for EV-A71 infection that could be consistently infected by the oral route. With optimum viral doses, the model invariably developed CNS infection and squamous epithelial lesions in the paws, skin and oral cavity that is strikingly reminiscent of HFMD. This model extends our existing knowledge on the viral cellular targets and pathogenesis of EV-A71 infection.

## Materials and Methods

### EV-A71 MAV Stock Preparation and Titration

The EV-A71 MAV used in our experiments was previously produced by serially passaging infected brains in 1-day-old ICR mice, and originally developed for a mouse model of EV-A71 encephalomyelitis [21]. Vero cells grown in Dulbecco’s modified Eagle’s growth medium (DMEM) supplemented with 5% fetal bovine serum were used to grow and titrate the MAV stock. Vero cell monolayers in 2% DMEM were infected at a multiplicity of infection of 0.01 CCID_50_, and the virus titer was determined by a standard micro-titration assay as previously described [21].

### Animal Infection Experiments

The protocol for all animal experiments was approved by the Faculty of Medicine Institutional Animal Care and Use Committee, University of Malaya (Ethics Reference No: 2014-02-14/PATHO/R/WKT). New-born hamsters were raised for our experiments from pregnant Syrian golden hamsters purchased from Monash University, Malaysia. Each group of new-born hamsters was housed together with their mothers until the end of the experiments, and provided with adequate autoclaved food and water in an air-conditioned room with 12 hours light/dark cycles. Since preliminary results showed that 2-week-old hamsters could be consistently infected by the oral route, we performed a 50% lethal dose (LD_50_) study on these animals. Six groups of hamsters (6 animals per group) were each orally-infected with 100 μl phosphate buffered saline (PBS) containing 10^5^, 10^4^, 10^3^, 10^2^, 10, and 1 CCID_50_ of MAV, respectively. The infective dose was delivered using a micropipette and without anesthesia. Mock-infected hamsters (2 per group) were kept separately and given PBS only. All animals were closely monitored every 3 hours during working hours for up to 14 days, for signs of infection that included weight loss, humped posture, ruffled fur and hind limb paralysis. A total of 17 moribund animals defined as paralysis of ≥2 limbs (or clinical score 4 [28]), were euthanized with isoflurane (1–4%) to minimize suffering (humane endpoints). Tissues from these animals were kept for pathological analysis. Animals found dead (n = 7) overnight were dissected to determine the cause of death but the tissues were not included in the analysis. After adequate 10% neutral-buffered formalin fixation for about 2 weeks and separating the entire skin from the carcass, sets of tissues comprising 20 standard tissue cross-sections which included oral cavity, brain, spinal cord, most internal organs, hind limb muscle and paws from each animal were prepared for routine processing. Separated skin tissues were entirely submitted for processing as well.

In a second experiment, 2-week-old hamsters (n = 8) were orally infected with 100 μl PBS containing 10^4^ CCID_50_ MAV to correlate pathological findings with viral titres. Two mock-infected animals were also included in the experiment as described above. Animals were monitored, sacrificed and tissue sets for routine processing collected from 4 infected animals as before. From the other 4 infected animals, serum and solid organs were collected for viral titration. In addition, oral washes and feces for virus titration were collected daily from all 8 infected animals.

### Light Microscopy

Formalin-fixed tissue sets (n = 21) were decalcified in 5% formic acid overnight followed by further formalin fixation for 2 days before routine processing for paraffin-embedding. Tissue sections, 4 μm thick, were stained with hematoxylin and eosin for light microscopy. Similar sections were prepared for immunohistochemistry (IHC) and *in situ* hybridization (ISH).

### IHC

IHC was performed using a modified immunoperoxidase technique as described previously [21]. Briefly, a monoclonal mouse anti-EV-A71 antibody (Light Diagnostics, UK) was applied onto tissue sections to incubate for 2 hours at room temperature followed by secondary antibody-horseradish peroxidase for 30 minutes at room temperature (Dako Real Envision, Denmark). The substrate chromogen 3,3’ diaminobenzidine tetrahydrochloride was then applied (Dako, Denmark), followed by hematoxylin counterstaining and mounting. For negative IHC controls, duplicate assays in which the primary antibody was replaced by mouse isotype control IgG1 or Tris buffered saline was done. EV-A71 infected mouse tissues [21] were used as positive controls. IHC specificity testing was done on mock-infected hamster tissues as well.

### ISH

A 603 bp PCR amplicon was first produced from the 5’ untranslated region of the EV-A71 genome using a pair of specific primers (forward primer GTCTCAGTTCCATTCATGTC and reverse primer CCTAGCAGGGTAATACTCGCTA). Digoxigenin (DIG)-labelled DNA probes were generated by incorporating DIG-11-dUTP (Roche, Germany) in a second PCR using the first amplicon as a template as previously described [16]. For the ISH procedure, after pre-treatment with 0.2 N HCL and proteinase K (100 μg/ml) digestion for 20 minutes at 37°C, tissue sections were hybridized with 50 μl of hybridization solution at 95°C for 10 minutes, followed by incubation for 16 hours at 42°C in a moist chamber. Hybridization was detected using anti-DIG-Alkaline-Phosphatase-conjugate (Roche, Germany) followed by nitroblue tetrazolium/5-bromo-4-chloro-3-indolyl phosphate (Roche, Germany) substrate to obtain a colour reaction. For negative ISH controls, duplicate assays that omitted probes were done. EV-A71 infected mouse tissues were used as positive controls.

### Virus Isolation from Tissues, Oral Washes and Feces

For virus isolation and titration, sera, brain, spinal cord, heart, spleen, hind-limb muscle and gastrointestinal tissues were collected from 4 animals in experiment 2. Extra care was taken to minimize cross-contamination of tissues at harvesting. Tissues were weighed and immediately frozen at -80°C for later use. Virus titration was performed with the micro-titration assay as for MAV stock using 4 replicates for each tissue sample after homogenization in PBS to obtain 10% (wt/vol) [21].

To obtain oral washes, 100 μl PBS was pipetted into the oral cavity a total of 6 times without anesthesia. The pooled oral washes were then stored at -80°C for processing later or immediately treated with chloroform (1:10), centrifuged at 3500 rpm, for 20 minutes at 4°C and the supernatant stored at -80°C until use [29]. Four or 5 fecal pellets expelled spontaneously from the anus during handling were collected from each hamster using sterile forceps, stored at -80°C to be processed later, or immediately processed as described for oral washes, then stored until use.

### Statistics

To determine statistical significance between viral titers, student t-test was performed using the software IBM SPSS Statistics version 22. The results were expressed as mean ± standard deviation. A *p* value of <0.05 was considered significant.

## Results

### LD_50_ Study

**Fig 1:**
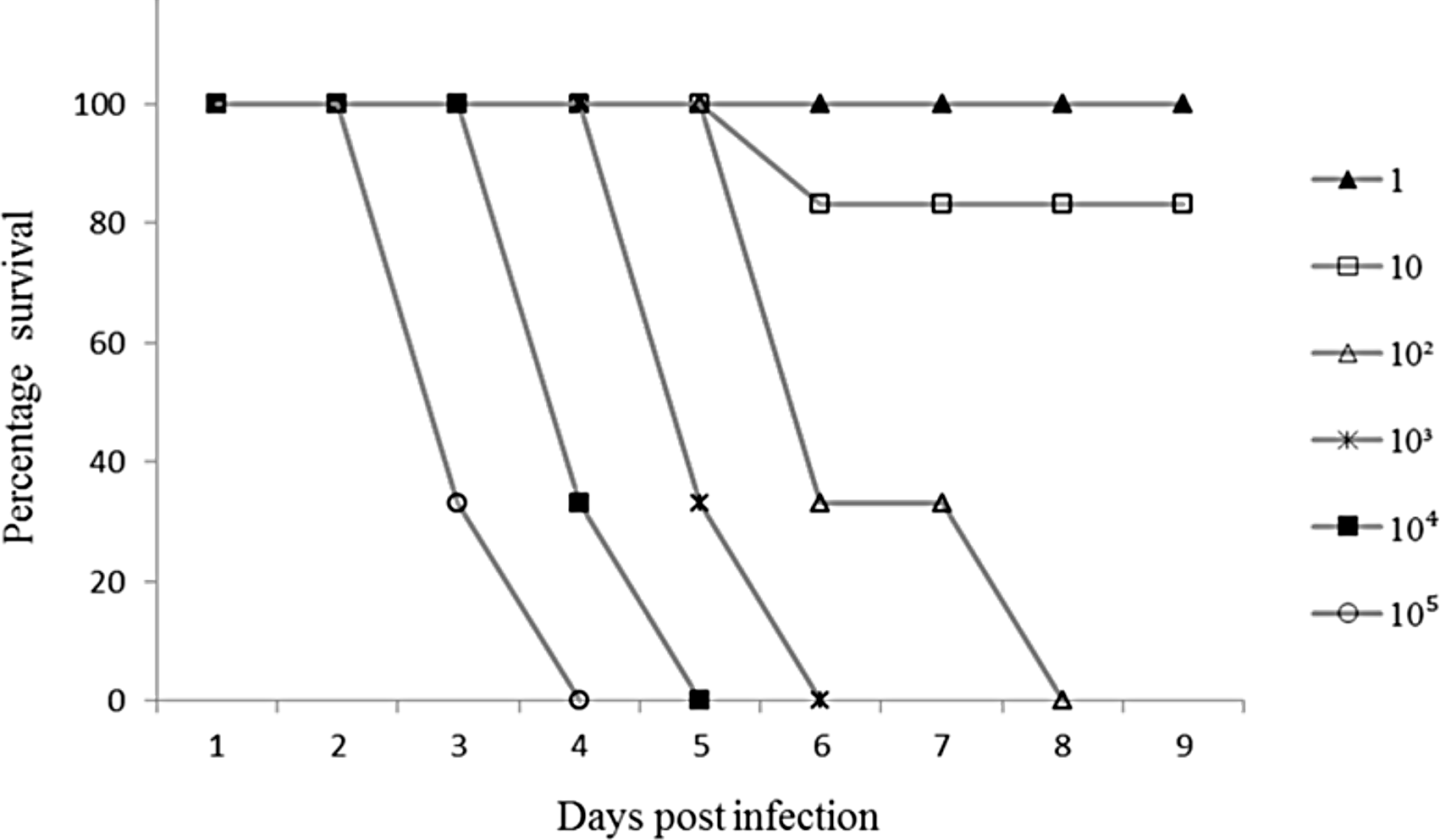
LD_50_ study: Survival graph of 2-week-old hamsters orally infected with six different viral doses (1−10^5^ CCID_50_). Each group comprised 6 animals. All animals infected with the 1 CCID_50_ dose survived.

**Fig 2:**
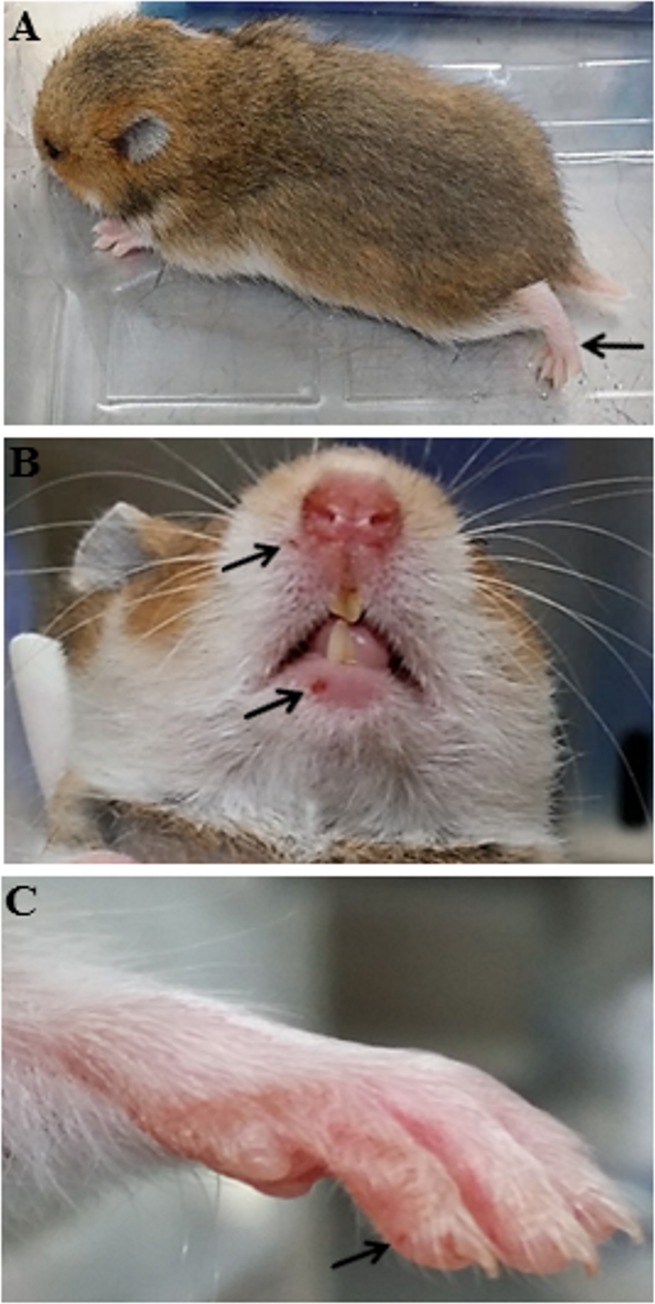
Infected hamster showing hind limb paralysis at day 4 post infection (A, arrow). Lesions on the lip (B, arrows) and paw (C, arrow) were observed in some animals.

Six groups of animals (n = 6 per group) were orally infected with serially-diluted viral suspensions ranging from 10^5^ to 1 CCID_50_. Overall, hamsters were susceptible to infection, and survival was dose-dependent (Fig 1). All animals given doses ≥10^2^ CCID_50_ died. The LD_50_ dose calculated using the method of Reed and Muench [30] was 25 CCID_50_. Animals consistently showed signs of infection such as hunchback posture, ruffled fur, weight loss and limb paralysis (Fig 2) before succumbing to infection between 3 to 8 days post infection (p.i). In the group given the low 10 CCID_50_ dose, about 16.7% died by day 8 p.i, displaying signs of infection and pathological findings similar to those infected with higher doses (Fig 1). The surviving animals in this group did not show any signs of infection and weight gain was consistent throughout the experiment. All the animals given the lowest 1 CCID_50_ dose appeared healthy except for 1 animal which developed hind limb paralysis at day 10 p.i. before recovering at day 14 p.i.

### Pathological Findings

**Fig 3:**
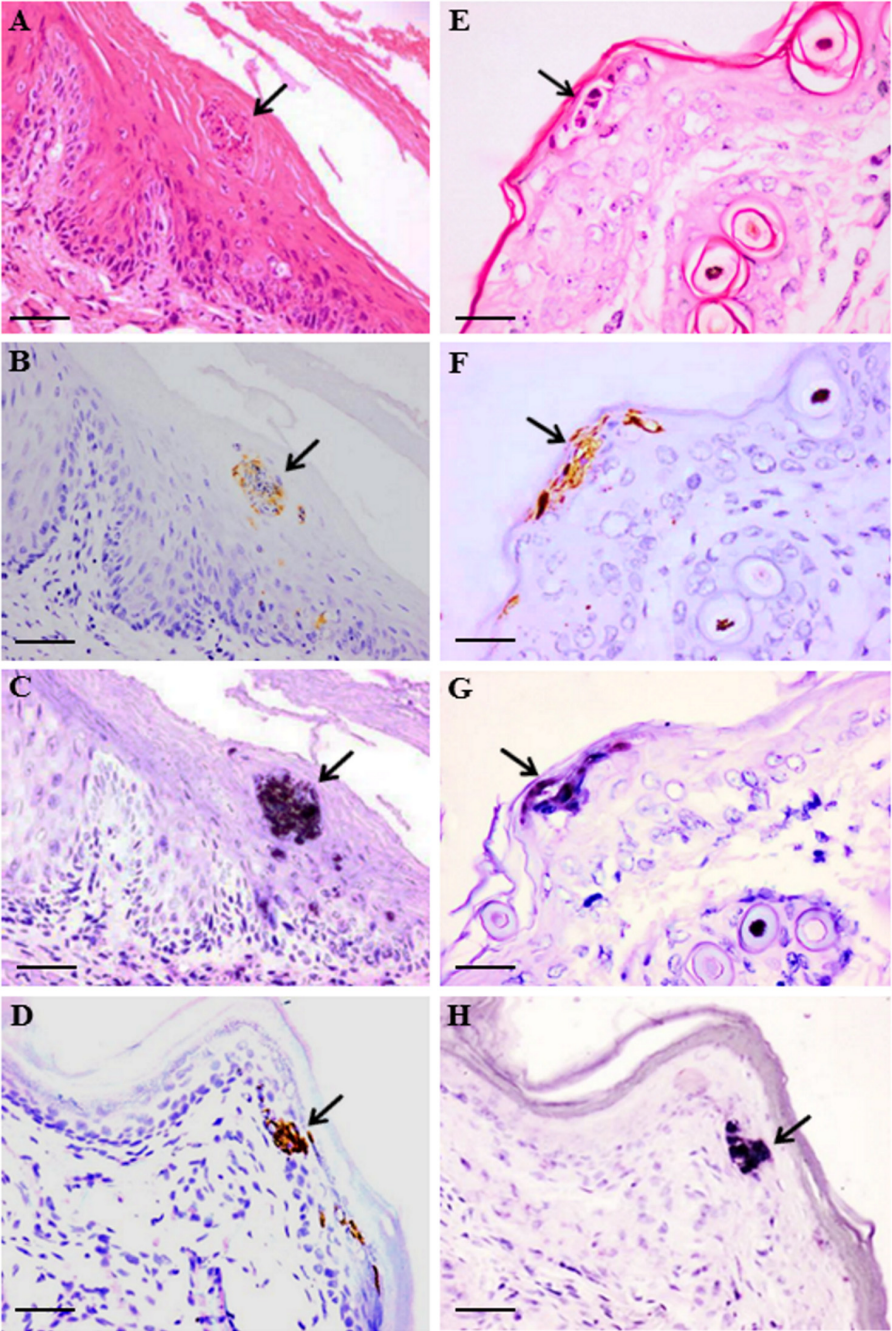
Pathological findings in respective serial tissue sections in EV-A71 infected hamsters at day 4 post infection. Focal squamous inflammatory lesion in the oral mucosa (A, arrow) and localization of viral antigens (B, arrow) and viral RNA (C, arrow) in the same lesion. In the skin epidermis, inflammatory lesion (E, arrow) with viral antigens (F, arrow) and viral RNA (G, arrow) were also found. Similarly, viral antigens (D, arrow) and viral RNA (H, arrow) were observed in the same lesion in the paw epidermis. Stains: Hematoxylin and eosin (A, E), immunohistochemistry with 3, 3’ diaminobenzidinetetrahydrochloride chromogen/hematoxylin (B, D, F), and in situ hybridization with nitroblue tetrazolium/5-bromo-4-chloro-3-indolyl phosphate/hematoxylin (C, G, H). Original magnification: 20x objective (A, B, C, D, H), 40x objective (E, F, G). Scale bars: 30μm (A, B, C, D, H), 15μm (E, F, G).

Macroscopic skin ulcers/lesions around the nose, lip and paw were visible in some of the infected hamsters given doses ≥10^2^ CCID_50_ (Fig 2). In all the 21 animals with tissues available for light microscopy, small discrete foci of viral antigens/RNA as shown by strong IHC and ISH positive signals, respectively, were localized to squamous cells in the epithelium covering the lips, tongue and other parts of the oral cavity (Fig 3B and 3C). Similarly, viral antigens/RNA were also found in the epidermis covering the paws (Fig 3D and 3H), limbs, head, neck, upper chest and pelvic areas (Fig 3F and 3G), including hair follicle germinal epithelium. These lesions generally showed minimal or mild inflammation, and some more superficial epidermal squamous lesions appeared vesicular. Viral antigens/RNA were also detected in esophageal squamous epithelium in 52% of animals but were very focal and less dense than in squamous epithelia elsewhere. Table 1 shows the distribution and relative density of viral antigens in squamous cells from various sites, CNS tissues and skeletal muscle fibres.

**Table 1:** Localization of viral antigens in various tissues in animals (n = 21) orally-infected with various CCID_50_* doses.

Dose/ Animal#	Oral** mucosa	Tongue mucosa	Esophageal mucosa	Skin epidermis	Paw epidermis	Brain stem	Spinal cord	Skeletal muscle***
**10**^**5**^ **CCID**_**50**_								
Animal 1	+	+	-	+	+	++	++	+++
Animal 2	+	+	-	+	+	-	++	+++
Animal 3	+	+	-	+	+	-	-	+++
Animal 4	+	+	+	+	+	-	-	+++
Animal 5	+	+	-	+	+	-	++	+++
Animal 6	+	+	+	+	+	++	++	+++
Animal 7	+	+	+	+	+	++	++	+++
Animal 8	+	+	-	+	-	++	++	+++
**10**^**4**^ **CCID**_**50**_								
Animal 1	+	+	-	+	+	-	++	+++
Animal 2	+	+	+	+	+	++	++	+++
Animal 3	+	+	+	+	+	++	++	+++
Animal 4	+	+	+	+	+	++	++	+++
Animal 5	+	+	+	+	+	-	++	+++
Animal 6	+	+	-	+	+	++	++	+++
**10**^**3**^ **CCID**_**50**_								
Animal 1	+	+	+	+	+	++	++	+++
Animal 2	+	+	NA	+	+	-	++	+++
Animal 3	+	+	-	+	+	++	++	+++
Animal 4	+	+	+	+	+	-	++	+++
**10**^**2**^ **CCID**_**50**_								
Animal 1	+	+	NA	+	+	++	++	+++
Animal 2	+	+	-	+	+	++	++	+++
**10 CCID**_**50**_								
Animal 1	+	+	NA	+	+	++	++	+++

*CCID_50_ = 50% cell culture infectious dose.

**Oral mucosa includes lip, buccal mucosa and mucosa from other parts of oral cavity except the tongue.

***Since, skeletal muscle fibres invariably showed the highest density of viral antigens, the highest semi-quantitative score of +++ was assigned to skeletal muscle.

+++ = >50% area positive

++ = 10–50%

+ = <10%

NA = not available

**Fig 4:**
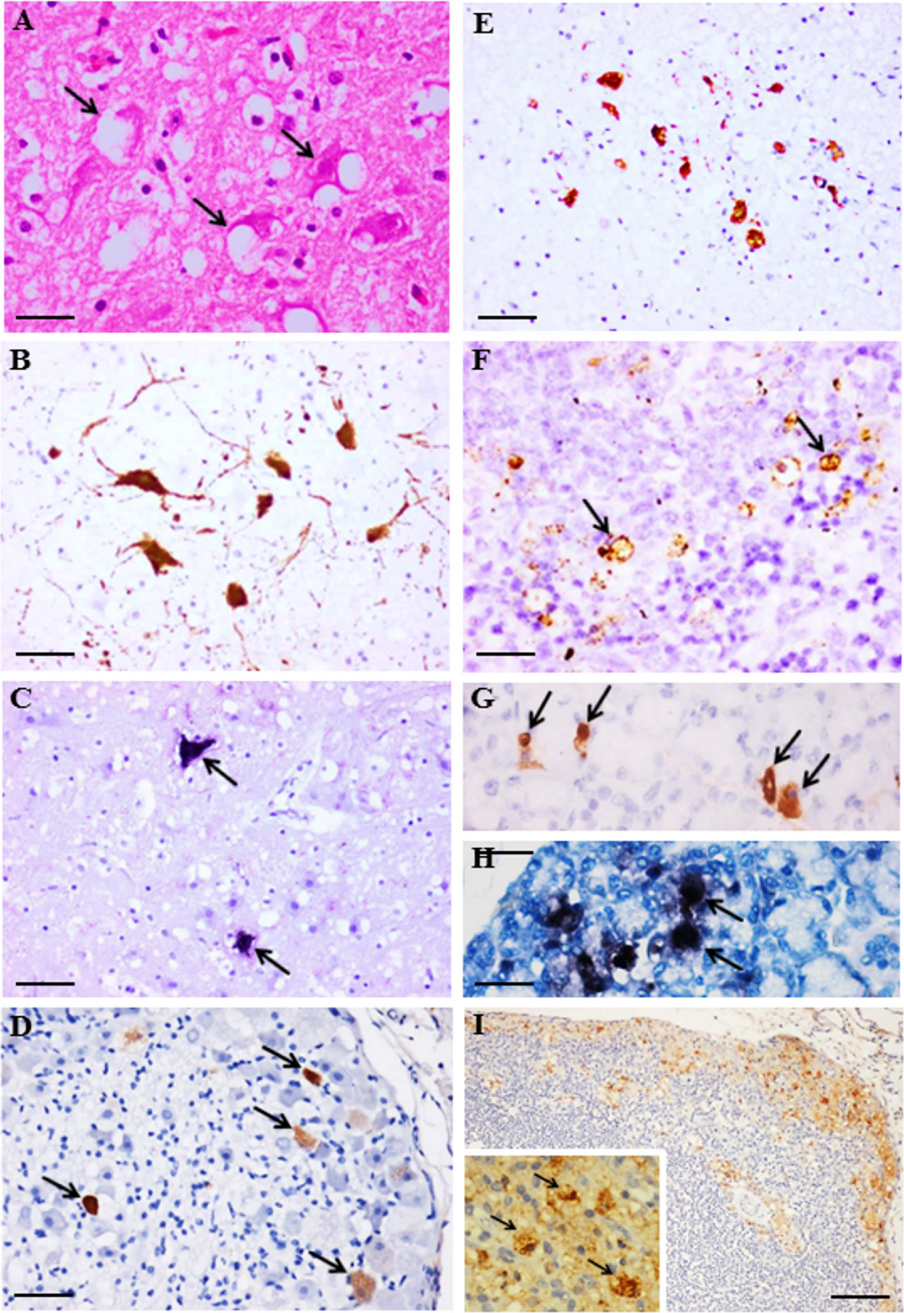
Pathological findings in CNS and non-CNS tissues from EV-A71 infected hamsters at day 4 post infection. Vacuolation and degeneration in brainstem neurons (A, arrows) that also demonstrated viral antigens (B) and RNA (C, arrows). Viral antigens were also detected in dorsal root ganglion (D, arrows) and spinal cord anterior horn cells (E). Viral antigens in the spleen (F, arrows), salivary gland acinar cells (G, arrows), and lymph node (I and inset, arrows), and viral RNA in the salivary gland acinar cell (H, arrows) were observed. Stains: Hematoxylin and eosin (A), immunohistochemistry with 3, 3’ diaminobenzidinetetrahydrochloride chromogen/hematoxylin (B, D, E, F, G, I), and in situ hybridization with nitroblue tetrazolium/5-bromo-4-chloro-3-indolyl phosphate/hematoxylin (C, H). Original magnification: 10x objective (I), 20x objective (B-E), 40x objective (A, F, G, H, I inset). Scale bars: 50μm (I), 30μm (B-E), 15μm (A, F, G, H, I inset).

In the CNS, although inflammation was minimal or mild, viral antigens/RNA were demonstrated in neuronal bodies and processes in the brainstem (62% of animals) and spinal cord anterior horn (90%) (Table 1), and in sensory ganglia (90%), mainly in dorsal root ganglia and occasionally in trigeminal ganglion (Fig 4B, 4D and 4E). Neurons in other parts of the CNS e.g. cerebral and cerebellar cortex, and other cells/tissues such as oligodendrocyte, astrocyte, ependyma, blood vessel, choroid plexus, meninges, peripheral nerve and autonomic ganglion were all IHC and ISH negative.

Focal acinar cells in the parotid salivary gland (71% of animals) (Fig 4G and 4H) and lacrimal gland (64%), scattered liver hepatocytes (71%), and rare stomach epithelial cells were positive for viral antigens/RNA. Subcapsular sinus macrophages and lymphoid cells in other parts of lymph nodes from the head, neck and other areas were positive for viral antigens only (77% of animals) (Fig 4I) while some lymphoid cells in the spleen were positive for both viral antigens and RNA (62%) (Fig 4F). Viral antigens/RNA were densest in inflamed skeletal muscle fibres found in all the animals (Table 1). Conversely, only very rare foci of viral antigens/RNA were found in the myocardium and smooth muscles of the intestine and stomach although this was observed in all the animals. There was no myocarditis or pulmonary edema. Lymphoid cells in the thymus, intestinal Peyer’s patches and mucosa, pancreas, kidney and lung were all negative for viral antigens/RNA.

### Virus Titration

**Fig 5:**
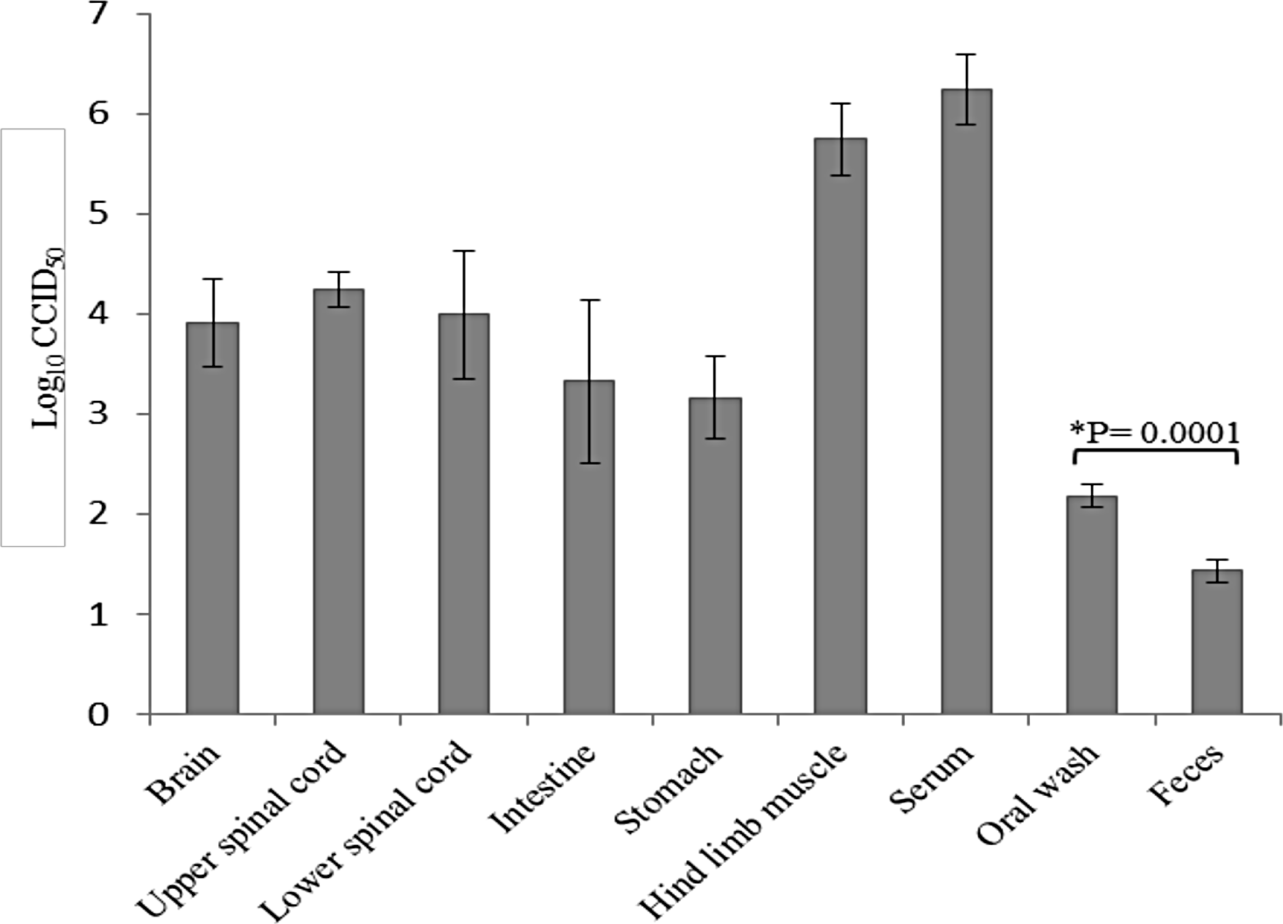
Viral titers in harvested tissues from EV-A71 infected hamsters (experiment 2; 10^4^ CCID_50_ dose). Virus titer is expressed as the mean log_10_ CCID_50_ ± standard error of mean per 10% tissue homogenates derived from 4 hamsters at day 4 post infection. The highest titters were obtained from serum, hind limb muscle and the CNS. Oral wash viral titer was significantly higher than feces (*P* = 0.0001).

Fig 5 shows virus titration in the sera, solid organs, oral washes and feces in the 4 animals from experiment 2. The highest titers were obtained in the serum, hind-limb muscles and the CNS. From day 4 p.i. onwards, virus could be isolated from oral washes and feces, with viral titers of 10^3^ CCID_50_ and 10^2^ CCID_50_, respectively.

## Discussion

Our unique hamster model could be consistently infected by the natural oral route employed by EV-A71 in human infections. With sufficiently high viral doses (≥10^2^ CCID_50_), the hamster invariably developed squamous lesions in the paw, skin and oral cavity, and encephalomyelitis that strikingly resembled complicated HFMD. It is intriguing that the involvement of oral cavity and paws corresponded so well with the mucocutaneous lesions in HFMD. The reasons are unknown, and further investigations using this model may be able to provide some clues. Based on our findings, the pathological basis for the oral lesions, skin rashes and vesicles in HFMD is viral squamous epitheliotropism, first suggested in a recent SCARB2 transgenic mouse which showed viral antigens in oral squamous epithelium and distal limb epidermis. However, these findings were only observed in subcutaneously-infected, neonatal transgenic mice, but not in older mice [23]. In our model, the strong positive IHC and ISH signals suggested active viral replication, and that viral cytolysis played an important role in the inflammation and necrosis of squamous cells. From its extensive involvement, the squamous epithelium in the oral cavity/tongue is likely to be an important primary viral replication site that contributes significantly to oral viral shedding. Moreover, active viral replication in salivary gland acinar cells may also contribute to viral shedding via its secretions. The relatively high viral titres obtained from oral washes in our model, ≥4 days p.i (Fig 5), suggested that these viruses represented viral progeny rather than the original inoculum. Viral replication in the human tonsil has been suggested as an important source site for primary viral replication contributing to viral shedding in oral secretions and feces [12]. Unfortunately, we were unable to confirm this since the hamsters do not have palatine tonsils. Interestingly, lacrimal gland acinar cell infection in this model raises the possibility that human tears may also be a source of shed virus.

As shown in the hamster epidermis, infection of human epidermal squamous cells probably occurs, leading to acute inflammation, and skin vesicle/rash formation. Hitherto, there is no data on human epidermal squamous cell infection [12] but virus has been cultured in fluid from skin vesicles [11,31,32]. Although EV-A71 associated skin lesions in mouse and monkey models have been reported before [13,19,25], only in the SCARB2 transgenic mouse model was epidermal involvement convincingly demonstrated [23]. In skin rashes/vesicles of herpes simplex and varicella zoster infections, viral antigens and virions in epidermal squamous cells have been previously demonstrated [33,34]. Based on the extent and severity of infection in the hamster model, the epidermis could be an important secondary viral replication site contributing significantly to viremia. Furthermore, if sufficient viable virus is shed from the skin, significantly person-to-person transmission via a cutaneous-oral route could possibly occur. While it may be assumed that skin infection itself resulted from viremia, further investigations are needed to exclude the possibility that direct percutaneous infection may occur after exposure to viruses in the environment.

In human studies and other animal models, we believe significant viral replication sites in the gastrointestinal tract mucosa have never been convincingly demonstrated [19,23,25,26,32,35]. Generally, in the hamster model we found no evidence of viral replication in gastrointestinal epithelium (except very focally in gastric epithelium) or any infection of pancreatic acinar cells and bile ducts, suggesting that virus shedding into the gastrointestinal tract from these tissues/organs may be insignificant. In fact, the main source of fecal virus may be the oral cavity, and to a much lesser extent, the esophageal mucosa and gastric epithelium. In human infections, it was reported that throat/oral swabs were more likely to be positive for virus than rectal swabs [11,36]. Of course, it is still possible that significant amounts of virus could be actively secreted into the digestive tract without mucosal viral replication but so far there is no evidence to support this. Hepatocyte infection may contribute to secondary viremia but virus may not be shed into the intestine via bile secretions. Similar to tonsillar crypt epithelium [12], it is also possible that the relatively thin oral/esophageal squamous epithelium upon infection could be portal for viral entry into the blood vessels in the subepithelial stroma.

The neuronotropism demonstrated mainly in the spinal cord and brainstem in our model confirmed findings in other animal models and human autopsy studies [16,20,21,23,24]. Based on these findings, it was suggested that retrograde axonal transport up peripheral and cranial motor nerves is an important route for viral entry into the CNS, resulting in early infection of motor neurons in the cord and brainstem. However, most of the animal models in these previous studies were infected by parenteral routes [21,23,24], hence the findings in our orally-infected model provide further corroborative evidence for this hypothesis. Viral antigens/RNA has been localized to sensory ganglia in our model and also in the transgenic mouse model [23]. So far, only inflammation in human dorsal root ganglion without viral antigens/RNA had been reported previously [16]. Dorsal root ganglia involvement may be due to viral transmission from infected anterior horn cells crossing into sensory nerves via the reflex arc or may even be the result of viral transmission up peripheral sensory nerve endings. These possibilities should be further investigated.

The very focal viral antigens/RNA found in hamster myocardium without significant myocarditis, have also been reported in mouse models [23,25]. Human viral myocarditis and/or viral antigens/RNA localization in the myocardium have not been unequivocally demonstrated [5,12,16,32,37]. The extensive skeletal muscle involvement in this hamster model is similar to most other mouse models, including a SCARB2 transgenic mouse [19]. So far, autopsy studies have not yielded any evidence of myositis nor shown localization of viral antigens/RNA [12,16,35] but there have been very few studies. Interestingly, rhabdomyosarcoma cells readily support viral replication *in vitro* [38,39].

Our hamster model, which was orally-infected by MAV produced by passaging virus in mouse brains, was serendipitously found to be more susceptible to infection than the original mouse model [21]. In all other previous mouse models, including a transgenic mouse, oral/intragastric inoculation did not consistently cause infection [21–23,25,26]. Similarly, oral infection in a pig model was inconsistent [40], and so far there has been no reports of orally-infected monkey models [13,15,20]. Based on IHC and ISH evidence of infection in the salivary and lacrimal glands, esophageal mucosa, gastric epithelium, and gastrointestinal smooth muscle, our hamster model demonstrated a wider tissue tropism than previously observed in animal models or human studies [12,13,15,16,19,21–23,25,32]. In our opinion, squamous cell infection in oral mucosa and epidermis has only been demonstrated in one other animal model, a transgenic mouse model [23]. In another transgenic mouse, and other mouse and monkey models, in which skin infection was investigated, squamous epitheliotropism was not convincingly demonstrated [13,19,25].

A relatively high degree of homology of SCARB2 receptors in the hamster and the mouse (90%) may explain why the former was also susceptible to MAV (S1 Fig) but there may be other reasons. The most consistent results were obtained in 2-week-old or younger hamsters, just like in other mouse models [19,21,26]. The reasons are not clear just as the reasons for a much higher incidence of complicated HFMD in children less than 5 years old are also not fully understood [7] but may due to immune system immaturity, relative availability of viral receptors or other unknown factors. In conclusion, we believe our orally-infected hamster is a good small animal model to further investigate viral pathogenesis, to model person-to-person transmission of EV-A71 infection, and to test the effectiveness of anti-viral drugs and vaccines [27].

## Supporting Information

S1 Fig Alignment of amino acid sequences of SCARB2 receptors in human, golden hamster and mouse. These sequences, human (*Homo sapiens*; accession no. KR709707), golden hamster (*Mesocricetu*s *auratus*; accession no. NM_001281557), and mouse (*Mus musculus*; accession no. NM_007644) were downloaded from Genbank and aligned using MEGA 6.06 software. The amino acid sequences conserved regions between all three sequences is about 82% and between hamster and mouse is about 90%.
(DOCX)

## Abbreviations

EV-A71: Enterovirus A71
HFMD: hand-foot-and-mouth disease
LD_50_: median lethal dose
CCID_50_: 50% cell culture infectious dose
p.i.: post infection
CNS: central nervous system
DMEM: Dulbecco’s modified Eagle’s growth medium
MAV: mouse-adapted virus
PBS: phosphate buffered saline
SCARB2: Scavenger Receptor Class B member 2
