# Supplementary material for: A Consistent Orally-Infected Hamster Model for Enterovirus A71 Encephalomyelitis Demonstrates Squamous Lesions in the Paws, Skin and Oral Cavity Reminiscent of Hand-Foot-and-Mouth Disease
Source: PLoS One. 2016 Jan 27;11(1):e0147463. doi: 10.1371/journal.pone.0147463 (PMC4729525; doi:10.1371/journal.pone.0147463)
Supplement: S1 Fig — These sequences, human (Homo sapiens; accession no. KR709707), golden hamster (Mesocricetus auratus; accession no. NM_001281557), and mouse (Mus musculus; accession no. NM_007644) were downloaded from Genbank and aligned using MEGA 6.06 software. The amino acid sequences conserved regions between all three sequences is about 82% and between hamster and mouse is about 90%. (DOCX) [file pone.0147463.s001.docx]

**Supporting Information**

**S1 Fig.** **Alignment of amino acid sequences of SCARB2 receptors in human, golden hamster and mouse.** These sequences, human (*Homo sapiens*; accession no. KR709707), golden hamster (*Mesocricetus auratus*; accession no. NM_001281557), and mouse (*Mus musculus*; accession no. NM_007644) were downloaded from Genbank and aligned using MEGA 6.06 software. The amino acid sequences conserved regions between all three sequences is about 82% and between hamster and mouse is about 90%.
